# Biochemical and Sociodemographic Correlates of Major Depressive Disorder in Patients With Chronic Kidney Disease Receiving Hemodialysis

**DOI:** 10.7759/cureus.43267

**Published:** 2023-08-10

**Authors:** Ashutosh Kumar, Apoorva Jain, Praveen Rikhari

**Affiliations:** 1 Psychiatry, Sarojini Naidu Medical College, Agra, IND; 2 Nephrology, Sarojini Naidu Medical College, Agra, IND

**Keywords:** ham-d, mdd, hemodialysis, depression, ckd

## Abstract

Introduction

Chronic kidney disease (CKD) is a chronic disabling illness with multiple physical and psychosocial consequences. A major treatment modality for CKD is renal replacement therapy in the form of hemodialysis. A bidirectional relationship between depression and CKD is proposed, as depressive symptoms lead to poorer outcomes in CKD and vice versa. This study aimed to determine the prevalence of major depressive disorder (MDD) in CKD patients on maintenance hemodialysis and find any sociodemographic, clinical, or biochemical correlates.

Material and methods

This was a cross-sectional study conducted at a tertiary care teaching hospital in north India. We used clinical interviews for diagnosing MDD according to the Diagnostic and Statistical Manual of Mental Disorders, Fifth Edition (DSM-5). The severity was quantified using Hamilton Depression Rating Scale (HAM-D) in those diagnosed with MDD.

Results

A total of 77 patients were included in the study. The prevalence of MDD in patients with CKD undergoing hemodialysis was 31.17%, much higher than in the general population. Most of those who were depressed had moderate to severe depression. Male gender and lower socioeconomic status were significantly associated with the diagnosis of MDD. Those undergoing dialysis less frequently or having more work days lost due to CKD were more likely to be depressed. Additionally, patients with MDD were found to have significantly lower blood hemoglobin levels, lower serum ionized calcium levels, and a lower urea reduction ratio compared to those who did not have MDD.

Conclusion

The high prevalence of MDD in patients with CKD undergoing hemodialysis suggests that clinicians should actively evaluate for depressive symptoms in this patient population and refer them to mental health services when required, especially those with the above-identified sociodemographic and biochemical risk factors.

## Introduction

Chronic kidney disease (CKD) is an important public health problem with significant physical and psychosocial consequences. The increasing number of people with hypertension and diabetes, along with increasing life expectancy, have additionally contributed to the rising prevalence of CKD. A recent multicenter study by the International Society of Nephrology’s Kidney Disease Data Centre (ISN-KDDC) reported the prevalence of CKD globally as 14.3% and in India as 16.8% [1]. Consequently, a large number of patients are on renal replacement therapy, including dialysis. In 2010, more than two million people worldwide received dialysis, which is expected to double by 2030 [2]. A 2018 estimate put the number of patients on maintenance dialysis in India at around 175,000, with a prevalence of about 129/million population [3]. This number is expected to increase with the increasing prevalence of CKD. Depression is common in chronic illnesses, and in CKD, it is the primary psychiatric illness [4,5]. A multitude of factors, like coming to terms with the diagnosis and its life-threatening implications as well as biochemical abnormalities, changes like an increased requirement for care, dependence on others, and functional and physical impairment due to the illness may together contribute to depressive symptoms in these patients [6,7]. A treatment modality like hemodialysis, which is a troublesome and unpleasant procedure by itself and must be done regularly, also has a role to play. Furthermore, in India, dialysis is primarily a private healthcare-driven initiative with significant expenses that add to the misery of the patients and families [3]. Studies show that depressive symptoms increase mortality risk and adverse medical outcomes like ED visits, hospitalizations, cumulative hospital days, cardiovascular events, dialysis withdrawal, and suicide in CKD patients [8-11]. They also lead to poorer quality of life and greater somatic and sexual complaints [12-14]. Therefore, a bidirectional relationship between CKD and depressive symptoms is evident, as CKD by itself predisposes patients to depressive symptoms, and depressive symptoms predict poor long-term outcomes in CKD patients. The biochemical parameters that may be associated with depression are less studied in the Indian population. Hence, to further the research in this field, this study aimed to estimate the prevalence of major depressive disorder (MDD) in CKD patients who are on hemodialysis and to find any sociodemographic, clinical, or biochemical correlates.

## Materials and methods

Study design

This was a cross-sectional study conducted in the dialysis unit of a government medical college in north India from February 2021 to April 2021 using convenience sampling. The sample size was calculated using the formula n = z2p(1-p)/d2, where n is the sample size, z is the statistic corresponding to a given confidence level, p is the expected prevalence, and d is precision. Keeping precision at 10%, confidence interval at 95%, and expected prevalence at 22.8% based on data from previously reported studies, the minimum effective sample size was calculated as 68 [5]. Those more than 18 years of age and undergoing maintenance hemodialysis for CKD were included in the study after written informed consent. A minimum of three months on hemodialysis was considered as the cut-off for inclusion in the study. The patients undergoing hemodialysis for conditions like acute kidney injury, substance poisoning, or malignancy and those who could not be interviewed due to altered sensorium or uncooperativeness were excluded. Patients with any previous psychiatric illness (prior to diagnosis of CKD) were also excluded.

The study was approved by the Institutional Ethical Committee of Sarojini Naidu Medical College, Agra (E.C. registration No.: ECR/1409/Inst/UP/2020) via letter Ref: SNMC/IEC/2021-13 dated February 12, 2021.

Procedure

Sociodemographic data, anthropometric measurements, and biochemical profiles of participants were collected. These included blood hemoglobin levels (in grams per deciliter), serum creatinine levels (in milligrams per deciliter), serum ionized calcium levels (in millimoles per liter), and serum albumin levels (in grams per deciliter). The average urea reduction ratio (in percentage) of the participants in the past three months was also calculated. The clinical interview was carried out by the same interviewer for all participants, and the diagnosis was made using the Diagnostic and Statistical Manual of Mental Disorders, Fifth Edition (DSM-5) criterion for MDD. The DSM-5 criteria for MDD include experiencing a depressed mood or loss of interest/pleasure in almost all activities for at least two weeks, along with five or more additional symptoms such as changes in appetite or weight, sleep disturbances, psychomotor disturbances, fatigue or loss of energy, feelings of worthlessness or excessive or inappropriate guilt, diminished ability to think or concentrate or indecisiveness and recurrent thoughts of death or suicide. These symptoms should cause significant distress or impairment in daily life [15]. In patients having MDD, the Hamilton Depression Rating Scale (HAM-D) was used to rate the severity of illness after taking permission from the Wiley group.

Assessment tools

DSM-5, published by the American Psychiatric Association, is the standard and extensively used set of criteria to diagnose various psychiatric disorders. It provides specific criteria for diagnosing different psychiatric disorders and is widely used for research [15]. HAM-D is the most widely used clinician-rated scale for assessing the severity of depressive illness. The 17-item HAM-D scale was used in the current study, with each item rated on a score of 0-4. The total score was categorized as 0-7 (normal), 8-13 (mild depression), 14-18 (moderate depression), 19-22 (severe depression), and ≥23 (very severe depression) [16].

Statistics

**Figure 1:**
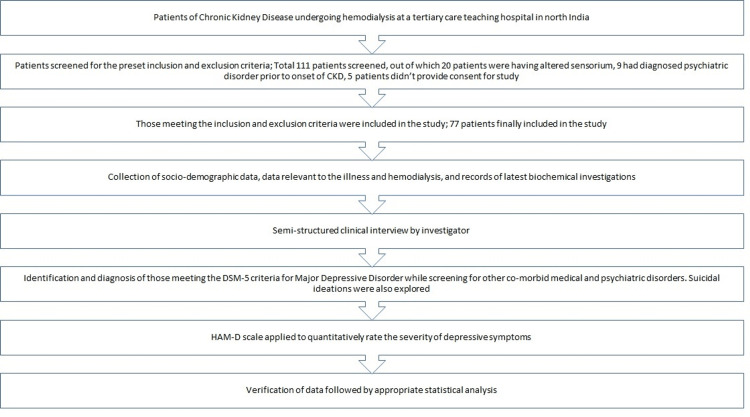
Overview of the study design.

Data were verified, tabulated, and entered into IBM SPSS version 27 (IBM Corp., Armonk, NY, USA) and analyzed. Chi-square/Fisher’s exact test was used for categorical variables. To evaluate the effect of continuous variables on the diagnosis of MDD, the Mann-Whitney U test and binomial logistic regression model were used. A p-value of 0.05 was considered to be statistically significant. An overview of study design is provided in Figure 1. 

## Results

A total of 111 individuals were screened, out of which 77 met the predefined inclusion and exclusion criteria. The age of participants was between 18 and 72 years, with a mean age of 43.41 years. The majority of participants were males (n=45, 58.44%), Hindus (n=66, 85.71%), and married (n=64, 83.12%) (Table 1).

**Table 1:** Sociodemographic characteristics of the sample population and their relation to major depressive disorder. MDD: Major depressive disorder; kg: Kilogram; m^2^: Square meter.

Parameters		Total (n=77)	Patients with MDD (n=24)	Patients without MDD(n=53)	P-value
Gender	Male	45 (58%)	18 (75%)	27 (51%)	0.03
Female	32 (42%)	6(25%)	26 (49%)		
Age (in years)	18-40	29 (38%)	9 (37.5%)	20 (38%)	0.81
≥40	48 (62%)	15 (62.5%)	33 (62%)		
BMI (in kg/m^2^)	<18.5	10 (13%)	2 (8%)	8 (15%)	0.16
18.5-24.9	43 (56%)	11 (46%)	32 (60%)		
≥25.0	24 (31%)	11 (46%)	13 (25%)		
Religion	Hindu	66 (86%)	21 (87.5%)	45 (85%)	0.96
Muslim	11 (14%)	3 (12.5%)	8 (15%)		
Family type	Joint	36 (47%)	10 (42%)	26 (49%)	0.55
Nuclear	41 (53%)	14 (58%)	27 (51%)		
Marital status	Married	64 (83%)	19 (79%)	45 (85%)	0.53
Unmarried/Widowed/Separated	13 (17%)	5 (21%)	8 (15%)		
Socioeconomic status (according to modified Kuppuswamy scale)	Upper	16 (21%)	6 (25%)	10 (19%)	<0.01
Upper middle/lower middle	33 (43%)	4 (17%)	29 (55%)		
Upper lower/lower	28 (36%)	14 (58%)	14 (26%)		
Sponsorship of dialysis	Free/Sponsored	44 (57%)	20 (83%)	24 (45%)	<0.01
Private	33 (43%)	4 (17%)	29 (55%)		

The mean time since diagnosis of CKD was 35.45 months, with 54 participants (70%) having the diagnosis for 12 months or more. Besides, the mean time since initiation of hemodialysis was 15.73 months, with 36 participants (47%) receiving hemodialysis for 12 months or more (Table 2). 

**Table 2:** Relationship of different variables to major depressive disorder in the sample population. CKD: Chronic kidney disease; HTN: Hypertension; DM: Diabetes mellitus; MDD: Major depressive disorder.

Parameters		Total (n=77)	Patients with MDD (n=24)	Patients without MDD (n=53)	P-value
Duration of CKD (in months)	<12	23 (30%)	6 (25%)	17 (32%)	0.72
	≥12	54 (70%)	18 (75%)	36 (68%)	
Time since initiation of hemodialysis (in months)	<12	41 (53%)	9 (37.5%)	32 (60%)	0.10
	≥12	36 (47%)	15 (62.5%)	21 (40%)	
Frequency of hemodialysis per month	<12	29 (38%)	13 (54%)	16 (30%)	0.04
	≥12	48 (62%)	11 (46%)	37 (70%)	
Percentage of work days lost per month	<75%	40 (52%)	8 (33%)	32 (60%)	0.02
	≥75%	37 (48%)	16 (67%)	21 (40%)	
Comorbidities (HTN and/or DM)	Present	55 (71%)	18 (75%)	37 (70%)	0.64
	Absent	22 (29%)	6 (25%)	16 (30%)	

Out of the total included participants, 24 (31.17%) met the DSM-5 criterion of MDD. Using the HAM-D scale, most were found to have moderate (n= 14, 58.33%) or severe depression (n=6, 25%). Three (3.9%) participants had suicidal ideations (Table 3). 

**Table 3:** Prevalence and severity of major depressive disorder in the sample population. DSM-5: Diagnostic and Statistical Manual of Mental Disorders, Fifth Edition; MDD: Major depressive disorder; HAM-D: Hamilton Depression Rating Scale.

Total number of subjects (n=77)		
DSM-5 Criterion for MDD met?	Yes	24 (31.17%)
	No	53 (68.83%)
Suicidal ideation present		3 (3.9%)
Number of subjects with MDD (n=24)		
HAM-D score in those having MDD	8-13 (mild depression)	3 (12.5%)
	14-18 (moderate depression)	14 (58.33%)
	19-22 (severe depression)	6 (25%)
	≥23 (very severe depression)	1 (4.17%)

We found that males were more likely to have MDD than females (p=0.03). Socioeconomic status was also noted to mediate the risk of having MDD (p<0.01), with post hoc analysis showing that lower socioeconomic classes (upper lower/lower) had a greater risk of MDD. The patients receiving free/sponsored dialysis had a statistically significant higher risk of having MDD (p<0.01) (Table 1). The frequency of hemodialysis per month and work days lost in a month also predicted being diagnosed with MDD. Those who underwent hemodialysis more than 12 times a month (three times a week) were less likely to be depressed. In contrast, those who underwent hemodialysis less frequently were more likely to be depressed (p=0.04). Similarly, those who had lost more than three-fourths of workdays per month due to CKD were more likely to be depressed (p=0.02). Other illness and dialysis-related parameters, including duration of illness, time since initiation of dialysis, and comorbidities, were not found to be significantly associated with the risk of MDD (Tables 1-2). Among biochemical parameters, the mean blood hemoglobin levels (p=0.02), mean serum ionized calcium levels (p<0.01), and mean urea reduction ratio (p=0.01) were significantly lower in patients with MDD compared to those without MDD (Table 4).

**Table 4:** Biochemical parameters and their relation to major depressive disorder in the sample population. MDD: Major depressive disorder.

Parameters	Patients with MDD (n=24)	Patients without MDD (n=53)	P-value
Blood hemoglobin (in grams per deciliter)	7.75±1.38	8.58±2.24	0.02
Serum creatinine (in milligrams per deciliter)	8.76±15.5	8.46±10.74	0.74
Serum albumin (in grams per deciliter)	3.15±0.66	3.37±0.39	0.49
Serum calcium (ionized) (in millimoles per liter)	0.82±0.06	0.99±0.05	<0.01
Average urea reduction ratio (in percentage)	39±4	48±2	0.01

The blood hemoglobin levels (p=0.03) and serum calcium levels (p=<0.01) continued to be significantly associated with the diagnosis of MDD after applying the binomial logistic regression model (Table 5).

**Table 5:** Binomial logistic regression model showing the biochemical parameter associated with major depressive disorder in the sample population. b_0 _:Regression constant; Exp (B): Exponentiation of the B coefficient; MDD: Major depressive disorder.

Parameters	Logistic Regression Coefficient	Standard Error	Z-statistic	Exp (B)	P-value
b_0 _	7.565	2.829	2.674	1928.952	0.01
Blood hemoglobin (in grams per deciliter)	-0.558	0.251	-2.221	0.573	0.03
Serum creatinine (in milligrams per deciliter)	-0.044	0.084	-0.518	0.957	0.62
Serum albumin (in grams per deciliter)	0.003	0.419	0.007	1.003	0.95
Serum calcium (ionized) (in millimoles per liter)	-3.075	1.388	-2.216	0.046	<0.01
Average urea reduction ratio (in percentage)	-1.592	177.3	-0.898	0.204	0.93

## Discussion

The strength of this study is the use of standardized and validated criteria in the form of DSM-5 to diagnose MDD based on clinical interviews conducted by a mental health professional. In contrast, most previous studies were based on clinician-rated or patient-rated screening questionnaires and were undertaken by non-mental health professionals who are comparatively less trained in diagnosing psychiatric disorders. The mean age of the participants was 43.41 years which is lower than that reported in previous studies from India. The lesser age may reflect a trend toward an increasing incidence of CKD in the younger age groups. The majority were males and married, similar to previous studies [17-19]. After interviewing the patients, the prevalence of MDD among the participants was found to be 31.17%, much higher than the prevalence in the general population (3.3%) [20]. Globally, the prevalence of depressive symptoms in patients undergoing dialysis ranges from 13.1% to 76.3% [21]. Indian studies report that 21.76%-83.5% of patients with CKD have depressive symptoms [17,19]. This wide variation in the range may be explained by the differing methodologies (screening questionnaire-based diagnosis vs. clinical interview-based diagnosis), with studies using clinical interviews for diagnosis giving lower prevalence estimates than studies utilizing screening questionnaires. This is further substantiated by a meta-analysis that reported that in patients with CKD undergoing maintenance dialysis, the summary prevalence of depression was 39.3% when evaluated by screening questionnaires and only 22.8% when evaluated by clinical interviews [5]. In our study, we also found that most of the patients with MDD were likely to have higher scores of HAM-D, suggesting more severe symptoms. This is understandable as the HAM-D scale also scores for various somatic symptoms of depression that overlap with the uremic symptoms commonly present in patients with CKD. In our study, we found that male sex (p=0.04), lower socioeconomic status (p=0.01), less frequent hemodialysis (p=0.04), and a greater percentage of work days lost per month (p=0.02) conferred a significantly increased risk of being diagnosed with MDD. A cross-sectional study conducted on the Chinese population between April 2009 to April 2010 found the male gender to be significantly associated with self-reported depressive symptoms [22]. On the other hand, other studies have found age, female gender, unemployment, low income, low education, nuclear family, being single, a longer time on maintenance hemodialysis, and comorbidities associated with depressive symptoms [23,24]. The findings of our study can be explained by the fact that most males are the primary earners of the family in India, and CKD reduces their potential to earn and fulfill social responsibilities resulting in depressive symptomatology. The finding related to those at a higher risk of MDD based on socioeconomic status suggests that people with lower socioeconomic status are more incapacitated because of CKD and suffer a greater financial impact of the illness and dialysis. A significantly higher proportion of MDD among patients receiving free or sponsored dialysis (p=0.01) reflects that other expenses and the loss of earning capacity contribute to MDD symptoms even after providing free or sponsored dialysis. The increased risk of MDD linked to a greater percentage of work days lost also substantiates the above inferences. The association of MDD with lesser frequency of dialysis reflects possible inadequate clearance of uremic toxins from the body and less robust adherence to treatment advice. We also did not find any significant association of depressive symptoms with the duration of CKD or time since initiation of hemodialysis, unlike some other studies [18,25]. We noted that lower blood hemoglobin levels (p=0.02), lower serum ionized calcium levels (p=0.01), and lower blood urea reduction ratio (p=0.03) were significantly associated with the diagnosis of MDD. The association with low hemoglobin might result from increased somatic symptoms and fatigue resulting from the effects of hemoglobin deficiency on multiple body systems. The low ionized calcium may indicate a vitamin D deficit known to cause depressive symptomatology [26]. The association with lower urea reduction ratio shows that less efficient dialysis might also contribute to MDD consequent to uncleared uremic toxins. This is also justified by the association of MDD with less frequent hemodialysis in the present study. A similar study found low blood hemoglobin levels to be significantly associated with depressive symptoms, while another study concluded that serum creatinine levels are not associated with depressive symptoms [18,27]. Another author reported lower serum albumin and not urea, calcium, or phosphate to be significantly associated with depression [26]. On the other hand, a systematic review found urea, hemoglobin, creatinine, and serum albumin to be associated with depressive symptoms [28].

Limitations

The limitations of the study include a small sample size and data from only one center. Moreover, the HAM-D may have overestimated the severity of depressive illness, as it takes into account somatic symptoms that overlap between CKD and MDD. Future studies with a longitudinal design and a larger number of patients from multiple centers could provide additional valuable insight. Also, vitamin D and vitamin B12 levels, which may be deranged in CKD and have been implicated in the pathophysiology of depressive illnesses, were not considered in the current study and should be explored.

## Conclusions

Our study demonstrates that MDD is much more prevalent in patients with CKD on hemodialysis than in the general population. Male gender, patients from lower socioeconomic strata, and those with more work days lost are more likely to be depressed, reflecting a greater impact of illness on them. This issue can potentially be mitigated by making healthcare services, especially dialysis services, more accessible, and by providing financial and occupational support to those affected by CKD and undergoing hemodialysis. We also found that patients with MDD had lower blood hemoglobin levels, lower serum ionized calcium levels, a lower urea reduction ratio, and less frequent hemodialysis, suggesting a need for more aggressive treatment. Thus, it may be prudent to identify and screen these susceptible patients for depressive symptoms and provide timely intervention to improve their quality of life and overall outcomes.

## References

[1] Ene-Iordache B, Perico N, Bikbov B (2016). Chronic kidney disease and cardiovascular risk in six regions of the world (ISN-KDDC): a cross-sectional study. Lancet Glob Health.

[2] Liyanage T, Ninomiya T, Jha V (2015). Worldwide access to treatment for end-stage kidney disease: a systematic review. Lancet.

[3] Jha V, Ur-Rashid H, Agarwal SK, Akhtar SF, Kafle RK, Sheriff R (2019). The state of nephrology in South Asia. Kidney Int.

[4] Kimmel PL, Thamer M, Richard CM, Ray NF (1998). Psychiatric illness in patients with end-stage renal disease. Am J Med.

[5] Palmer S, Vecchio M, Craig JC (2013). Prevalence of depression in chronic kidney disease: systematic review and meta-analysis of observational studies. Kidney Int.

[6] Song MK, Ward SE, Hladik GA, Bridgman JC, Gilet CA (2016). Depressive symptom severity, contributing factors, and self-management among chronic dialysis patients. Hemodial Int.

[7] Katon WJ (2003). Clinical and health services relationships between major depression, depressive symptoms, and general medical illness. Biol Psychiatry.

[8] Farrokhi F, Abedi N, Beyene J, Kurdyak P, Jassal SV (2014). Association between depression and mortality in patients receiving long-term dialysis: a systematic review and meta-analysis. Am J Kidney Dis.

[9] Hedayati SS, Grambow SC, Szczech LA, Stechuchak KM, Allen AS, Bosworth HB (2005). Physician-diagnosed depression as a correlate of hospitalizations in patients receiving long-term hemodialysis. Am J Kidney Dis.

[10] Boulware LE, Liu Y, Fink NE, Coresh J, Ford DE, Klag MJ, Powe NR (2006). Temporal relation among depression symptoms, cardiovascular disease events, and mortality in end-stage renal disease: contribution of reverse causality. Clin J Am Soc Nephrol.

[11] Kurella M, Kimmel PL, Young BS, Chertow GM (2005). Suicide in the United States end-stage renal disease program. J Am Soc Nephrol.

[12] Leinau L, Murphy TE, Bradley E, Fried T (2009). Relationship between conditions addressed by hemodialysis guidelines and non-ESRD-specific conditions affecting quality of life. Clin J Am Soc Nephrol.

[13] Pisoni RL, Wikström B, Elder SJ (2006). Pruritus in haemodialysis patients: International results from the Dialysis Outcomes and Practice Patterns Study (DOPPS). Nephrol Dial Transplant.

[14] Bornivelli C, Aperis G, Giannikouris I, Paliouras C, Alivanis P (2012). Relationship between depression, clinical and biochemical parameters in patients undergoing haemodialysis. J Ren Care.

[15] American Psychiatric Association (2013). Diagnostic and Statistical Manual of Mental Disorders (DSM-5-TR).

[16] Hamilton M (1960). A rating scale for depression. J Neurol Neurosurg Psychiatry.

[17] Nelson V, Gopalakrishnan S, Rakesh PS, Simon S (2016). Depression among dialysis patients attending a tertiary care hospital in Kerala, Southern India. J Nephrol Soc Work.

[18] Gupta S, Patil NM, Karishetti M, Tekkalaki BV (2018). Prevalence and clinical correlates of depression in chronic kidney disease patients in a tertiary care hospital. Indian J Psychiatry.

[19] Vermani A, Marwale A, Gokani N (2020). Psychiatric morbidity in end-stage renal disease patients' on dialysis. MGM J Med Sci.

[20] Sagar R, Dandona R, Gururaj G (2020). The burden of mental disorders across the states of India: the Global Burden of Disease Study 1990-2017. Lancet Psychiatry.

[21] Tian N, Chen N, Li PK (2021). Depression in dialysis. Curr Opin Nephrol Hypertens.

[22] Hou Y, Li X, Yang L (2014). Factors associated with depression and anxiety in patients with end-stage renal disease receiving maintenance hemodialysis. Int Urol Nephrol.

[23] Shirazian S, Grant CD, Aina O, Mattana J, Khorassani F, Ricardo AC (2017). Depression in chronic kidney disease and end-stage renal disease: similarities and differences in diagnosis, epidemiology, and management. Kidney Int Rep.

[24] Goh ZS, Griva K (2018). Anxiety and depression in patients with end-stage renal disease: impact and management challenges - a narrative review. Int J Nephrol Renovasc Dis.

[25] Kumar V, Khandelia V, Garg A (2018). Depression and anxiety in patients with chronic kidney disease undergoing hemodialysis. Ann Indian Psychiatry.

[26] Hoang MT, Defina LF, Willis BL, Leonard DS, Weiner MF, Brown ES (2011). Association between low serum 25-hydroxyvitamin D and depression in a large sample of healthy adults: the Cooper Center longitudinal study. Mayo Clin Proc.

[27] Aggarwal HK, Jain D, Dabas G, Yadav RK (2017). Prevalence of depression, anxiety and insomnia in chronic kidney disease patients and their correlation with the demographic variables. Pril (Makedon Akad Nauk Umet Odd Med Nauki).

[28] Gebrie MH, Ford J (2019). Depressive symptoms and dietary non-adherence among end stage renal disease patients undergoing hemodialysis therapy: systematic review. BMC Nephrol.

